# Comparative effects of quercetin and hydroalcoholic extract of *Otostegia persica* boiss with atorvastatin on atherosclerosis complication in male wistar rats

**DOI:** 10.1002/fsn3.1136

**Published:** 2019-08-16

**Authors:** Ali Parvin, Parichehreh Yaghmaei, Mehdi Noureddini, Sayyed Ali Haeri Roohani, Saeed Aminzadeh

**Affiliations:** ^1^ Department of Biology, Science and Research Branch Islamic Azad University Tehran Iran; ^2^ Physiology Research Center Kashan University of Medical Sciences Kashan Iran; ^3^ Gametogenesis Research Ceneter Kashan University of Medical Sciences Kashan Iran; ^4^ Bioprocess Engineering Research Group National Institute of Genetic Engineering and Biotechnology Tehran Iran

**Keywords:** atherosclerosis, atorvastatin, hydroalcoholic extract, otostegia persica Boiss, quercetin

## Abstract

The use of herbal remedies is significantly considered in the atherosclerosis treatment, reduction of fatty elements, and prevention of activity of oxidative stress factors. The present study was conducted on 48 rats in 6 groups. The experimental and sham groups were fed with 2% cholesterol for 40 days; and experimental groups were separately treated by atorvastatin, quercetin, and hydroalcoholic extract for 4 weeks. After treatment procedure, some serum factors such as low‐density lipoprotein (LDL), total cholesterol (TC), malondialdehyde (MDA), and reactive oxygen species (ROS) were evaluated. Serum levels of LDL, TC, MDA, and ROS were significantly lower in experimental groups than sham group (*p* < .01). There was a significant decrease in serum MDA levels of these two groups in comparison with the atorvastatin‐treated group (*p* < .05). Blood pressure parameters were decreased in treated with quercetin and hydroalcoholic extract in comparison with the sham group (*p* < .05). Quercetin and hydroalcoholic extract similar to atorvastatin could decrease serum lipids [except high‐density lipoprotein (HDL)], oxidative stress factors, aorta contraction, weight gain, and blood pressure. These reagents improved the vascular structure and prevented the plaque formation.

## 1 INTRODUCTION

Atherosclerosis, the most common heart disease, is an abnormal accumulation, oxidation, and modification of lipids that mostly causes the chronic inflammation in walls of arteries, vessel stenosis, and lumen obstruction and reduces the blood flow to the myocardial muscle (Amom, Azman, Ismail, Shah, & Arshad, 2011). Many factors can contribute to the development of atherosclerosis such as hypercholesterolemia, diabetes, smoking, obesity, hypertension, and inactivity (Insull, 2009). About 17.5 million people died of cardiovascular diseases by 2005; and the highest cardiovascular mortality rate was reported with 434 deaths in men and 235 in women in 2010 with 100,000 people per year in the eastern Europe (Herrington, Lacey, Sherliker, Armitage, & Lewington, 2016; Rodriguez‐Saldana, Rodriguez‐Flores, Cantu‐Brito, & Aguirre‐Garcia, 2014).

The CHD mortality increased to approximately 120% in men and 137% in women in developed countries during 1990 to 2020 (Sanchis‐Gomar, Perez‐Quilis, Leischik, & Lucia, 2016). Fatty streaks are among first damages of the atherosclerosis. Most of these fatty streaks are destroyed, but some of them are converted into plaques that make the lumen narrow and the vessel stiff (Rafieian‐Kopaei, Setorki, Doudi, Baradaran, & Nasri, 2014). Blood flow of arterial areas, which are susceptible to atherosclerosis, puts pressures on endothelium cells leading to an increase in molecules (Adhesion molecules) in endothelium cells, thereby increasing the ability of cells to adhere to the environment and other cells (Yurdagul, Finney, Woolard, & Orr, 2016). Furthermore, more inflammatory molecules are produced by endothelial cells in this high‐pressure environment. In this regard, hemodynamic pressures and increased blood lipoproteins activate the defective cycle, spread the disease, and cause the aneurysm, thrombosis, ischemia, and even myocardial infarction (Upadhyay, 2015).

The atheroma is the nonsymmetrical thickening of intima‐media layers of medium and large vessels. The inter‐duct of vessels is narrowed by thickening the intima layer (Lorenz, von Kegler, Steinmetz, Markus, & Sitzer, 2006). A mixture of all these tissue changes can be seen in patients with severe and symptomatic types of lesion. Accordingly, the lesion begins from the formation of fatty streaks at the intima layer containing bubble cells in young people, and it then transforms a fibroatheromatous plaque, a fibrous plaque and a complicated plaque, respectively (Agius, 2007). The inflammatory and immune cells, which enter the lesion from the blood constitute, are main parts of lesion (Fishbein & Fishbein, 2015). Endothelium and smooth muscle cells are the remaining cells in the structure of this lesion. Foam cells appear in the atheroma center that is limited by a cap composing of muscle cells and a collagen‐rich matrix (Nadkarni et al., 2007). The lymphocyte T cells, macrophages, and mast cells, which penetrate into the lesion, are abundant on the sides of lesion, its prominence extends to the inner healthy part of the vessel (and creates a layer around the lesion). This is the place where the atheromatic lesion develops. Hypercholesterolemia triggers the activation of endothelial cells in arteries and the retention of lipoproteins. The low‐density lipoprotein (LDL) at the intima layer is a major contributor to the onset of atherosclerosis (Wang‐Michelitsch & Michelitsch, 2015). The oxidation of these lipoproteins by free radicals, which are produced from macrophages, and their oxidative enzyme changes in the intima ultimately produce oxidized lipids. These oxidized lipoproteins release phospholipids that activate endothelial cells, especially in vessel areas under the hemodynamic pressure. Oxidized LDLs stimulate inflammatory reactions, attract monocytes, and mobilize and reproduce macrophages from absorbed monocytes (Tsoupras, Lordan, & Zabetakis, 2018). These inflammatory reactions occur to eliminate oxidized LDLs. In this period, oxidized fats activate more macrophages and exacerbate their oxidation phenomena. In the presence of hypercholesterolemia, inflammatory responses, which start to neutralize effects of oxidized LDLs, cannot complete their functions, and the oxidation of lipoproteins, and more inflammation in intima remain instead of the inflammation cycle (Panth, Paudel, & Parajuli, 2016). T‐lymphocytes emergence at early stages of atherosclerotic lesions as a defective cycle exacerbated inflammatory reactions. Macrophage cells and smooth muscle cells, which creep with oxidized lipoproteins, and their cytoplasms are filled with droplets containing cholesterol esters and thus creates the appearance of foaming cells (Al Batran, Al‐Bayaty, Al‐Obaidi, Hussain, & Mulok, 2014). Several factors, including platelets and adenosine diphosphate (ADP), serotonin, thromboxane, thrombospondin, and fibrinogen, are activating or restricting in the creation of plaques in the vessel (Sangkuhl, Shuldiner, Klein, & Altman, 2011).

The use of atorvastatin, which is capable of reducing cholesterol and LDL levels, is a method for treatment of atherosclerosis drug. Atorvastatin prevent the creation of foam cells in vessel walls of endothelial and smooth muscle cells. Most of the cytokines, which cause the inflammatory activity, are produced by these cells in the atheroma (Jorge, Almeida, Ozaki, Jorge, & Carneiro, 2005). Lipoprotein oxidation resulting from free radicals, enzymatic changes in intima, production of active lipids, and malondialdehyde (MDA) and reactive oxygen species (ROS) Enzymatic activity is associated with increased inflammation. Meanwhile, oxidized lipoproteins and phospholipids are mainly released resulting in activation of endothelial cells, especially in areas of arteries that are activated by the hemodynamic pressure (Panth et al., 2016).

Special attention has been paid on the use of herbal remedies in the treatment of atherosclerosis and their effects such as reducing fatty elements and preventing the activity of oxidative stress factors as they play curative roles in the atherosclerosis. The therapy may include the use of traditional medicines and herbal drugs. Flavonoids can lower cholesterol and efficiently prevent the creation of plaques in arteries' intima layers due to its antioxidant activity (Habauzit & Morand, 2012).

The *Otostegia persica *Boiss family (herbaceous and perennial) is lamiaceae and can be found in Iran, India, and Pakistan. *Otostegia persica *Boiss is reputed for treating arthritis, gastric discomfort, diabetes, regulating hyperlipidemia and hypertension. It also has antibacterial and antioxidant activity. Several types of flavonols (Flavonoids derivatives) including kaempferol, alpha‐pinene, β‐sitosterol, isovitexin, morin, and quercetin have been diagnosed from the *O.  persica *Boiss (El‐Beltagi & Ahmed, 2016; Sadeghi, Akaberi, & Valizadeh, 2014). The properties of this plant are such as antioxidant, antimicrobial, anti‐diabetic, anti‐inflammatory, anti‐hypertensive, and hepatoprotective (Safaeian, Yaghoobi, Javanmard, & Ghasemi‐Dehkordi, 2017). The present study aimed to conduct a comparative evaluation of effects of quercetin and hydroalcoholic extract of *O. persica* Boiss with atorvastatin on the complication of atherosclerosis: weight, serum lipids, and oxidative stress (MDA & ROS) changes, histological aortic changes, aorta contraction (to KCl 60 mM), and blood pressure parameters (systolic, diastolic, mean arterial, and pulse pressure) changes at male Wistar strain rats.

## 2 MATERIALS AND METHODS

### 2.1 Animals

All animal tests were approved by the Ethics Committee of Islamic Azad University of Tehran and were performed in accordance with National Institute of Health Guide for the Care and Use of Laboratory Animals. The research was conducted on 48 male Wistar strain rats in Kashan Medical Education Research Center. The rats were kept at 22 ± 2°C under a 12‐hr light–dark cycle with a relative humidity of 45%–60%. The laboratory was based on ethical code set by the Ministry of Health of Iran. At the beginning of experiment, rats with weights of nearly 180 gr were randomly classified into 6 eight‐rat groups.

### 2.2 Experimental protocol

Negative control group: It includes rats, which were kept under standard laboratory conditions for 68 days, when no activity was performed and they were intact.

Positive control group: It includes rats, which were kept under standard laboratory conditions for 40 days, and then they daily received 1 mg/kg saline water through gavage for 28 days (Amna, Uzma, Umme, Farha, & Ambreen, 2016).

Sham group: It includes rats which were on a high‐fat diet with cholesterol (2% each day) for 40 days, and then they daily received 1 mg/kg saline water through gavage for 28 days (Amna et al., 2016).

Treatment group 1: It includes rats, which were on a high‐fat diet with cholesterol (2% each day) for 40 days, and then they daily received 40 mg/kg atorvastatin drug through gavage for 28 days (Jones et al., 2003).

Treatment group 2: It includes rats, which were on a high‐fat diet with cholesterol (2% each day) for 40 days, and then they daily received 25 mg/kg quercetin through gavage for 28 days (Zarei, Ashtiyani, Taheri, & Rasekh, 2014).

Treatment group 3: It includes rats, which were on a high‐fat diet with cholesterol (2% each day) for 40 days, and then they daily received 25 mg/kg hydroalcoholic extract of *O. persica* Boiss through gavage for 28 days (Zarei et al., 2014).

#### 2.2.1 Atherosclerosis in rats

Cholesterol 2% (Merck co of Germany; Product code: 103672) gavage and a high‐fat diet containing cholesterol 2%, carbohydrate 40%, lipid 15%, protein 40%, and 3% fiber were used for 40 days to create atherosclerosis and create atheroma plaque in rats (Amna et al., 2016).

Rats with ketamine–xylazine were anesthetized and blood samples were taken from rats' hearts after twenty‐eight days of treatment with effective doses of atorvastatin, quercetin, and hydroalcoholic extract of *O. persica* Boiss in three separate groups. Oxidative stress factors such as MDA, ROS, and serum lipids including LDL, high‐density lipoprotein (HDL), total cholesterol (TC), and triglycerides (TG) were then measured. Furthermore, a part of pectoral aorta of rats was put into formalin 3.7% for the histological analysis; and a part was placed in Krebs solution (KS) (Sigma K3753) for measurement of aortic contraction. At the beginning and end of any stage of experiment, the rats were weighed. Finally, blood pressure parameters (systolic, diastolic, mean arterial, and pulse pressure) were measured.

### 2.3 Histological preparation

**Figure 1:**
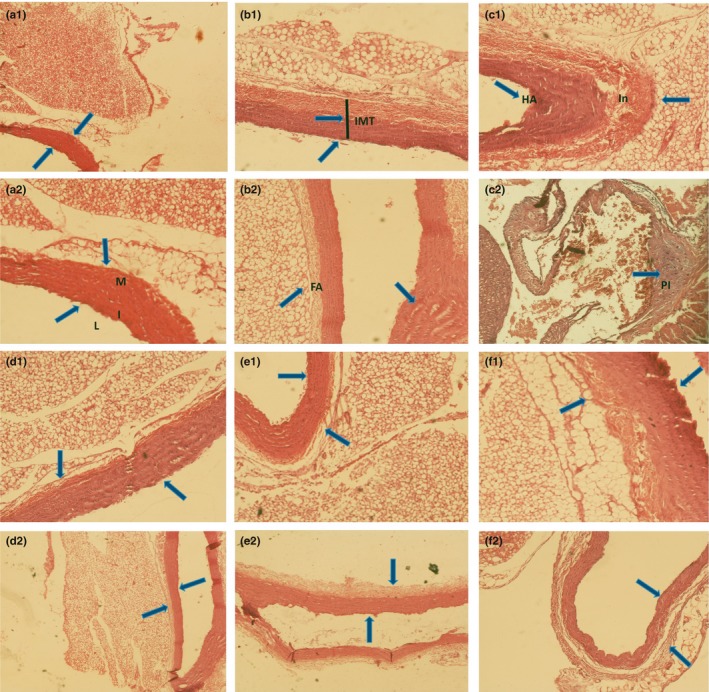
Histological aorta textures. a1 and a2 textures including rats aorta which were kept in standard laboratory conditions for 68 days (control group), (L = lumen, I = intima, *M* = media). b1, b2, c1 and c2 Textures including rats aorta that were on a high‐fat diet with cholesterol (2% each day) for 40 days and then in 28 days, daily received 1 mg/kg saline water. (IMT = Intima‐Media Thickness, FA = Fat Accumulation, Pl = Plaque, In = Inflammation, HA = Hemolysis Area). d1 and d2 Textures including aorta of rats that received atorvastatin through gavage. e1 and e2 Textures including aorta of rats that received quercetin through gavage. f1 and f2 Textures including aorta of rats that received hydroalcoholic extract of *Otostegia persica* Boiss through gavage

The aorta artery was isolated and placed in formalin 3.7%, and textures for dehydration were moved into the tissue processor after a few days; and the dehydration and clarification were carried out. We used ethanol 70%, grade 1, for 2 hr and ethanol 70%, grade 2, for 2 hr and ethanol 90%, grade 1, for 2 hr and ethanol 90%, grade 2, for 2 hr and ethanol 96%, grade 1, for 2 hr and xzilon 45%, grade 1, for 1 hr and xzilon 45%, grade 2, for 1 hr, respectively, and finally they were put in a solution of paraffin for 2 hr. Small micro‐templates containing paraffin and aorta tissue were then prepared, and then a microtome was used for 5 micrometers cuts of aortic tissue. Hematoxylin and eosin (H&E) staining was used for the microscopic study (Kim et al., 2017) (Figure 1).

### 2.4 Soxhlet extraction (SE)

*Otostegia persica* Boiss plant with a Herbarium IAUH‐000014912 was collected from Jiroft City of Kerman province and was confirmed by the Ibn Sina research Center of Science and Research Branch, Islamic Azad University of Tehran, Iran.

In short, 40 g of powdered leaves of *Otostegia persica* Boiss was placed in a soxhlet apparatus containing 80% methanol; and the hydroalcoholic extraction was extracted at several stages for 18 hr. After the evaporation of alcohol, a part of extract was used to extract quercetin. Water–alcohol 80% was used for several periods of flushing, and it was replaced in soxhlet; and the chloroform solution was isolated after 12 hr (Sharifi, Mahernia, & Amanlou, 2017).

### 2.5 Estimation of malondialdehyde, reactive oxygen species, and biochemical parameters

Amounts of MDA and ROS were measured using MDA and ROS Nalondi kits (Product Code: NS‐15022, Navandsalamat co). In both kits, instructions and bases were the same according to ELISA (enzyme‐linked immuno sorbent assay) sandwich type. In short, Kit sinks were covered with an initial antibody against the desired antigen (MDA, ROS). By adding the desired antigen, the next step was to add a secondary antibody that bound to the antigen. The enzyme in H.R.P was subsequently added and it stuck to the end of the secondary antibody. Washing out was the next step. The chromogenic solutions containing the HRP enzyme substrate were then added. Oxygenated water (H_2_O_2_) was exposed to H.R.P and converted into hydroxide radicals. Subsequently, it was exposed to a reagent that oxidized and produced color. In the next step, the production of color was stopped by stopping solution of sulfuric acid, and the continuation of the reaction was prevented. The color was studied with the ELISA Reader machine, the start‐fax‐2100 series manufactured by the United States, and was detected at 505 nm. The optical density specimen (O.D) and the sample concentration were determined based on the color. The more the color existed, the greater the light absorption was seen (Kumar, Kesari, & Behari, 2010; Olia, Ansari, Yaghmaei, Ayatollahi, & Khalkhali, 2017). In addition, serum lipids were also measured using instructions of catalogs of their kits.

### 2.6 Preparation and contraction measurement of rats' aorta

Rats were anesthetized and their pectoral aorta were isolated and placed in Krebs solution (KS) consisting of the following composition (in mM): NaCl 118, NaHCO_3_ 25, KH_2_PO_4_ 1.2, KCl, 4.7 MgSO_4_ 1.2, CaCl_2_ 2.5 and glucose 11 and oxygenated (4°C and pH 7.4) for 10 min (Qin et al., 2014).

The 4‐mm rings were then separated from the pectoral aorta and placed in a tissue bath containing a Krebs solution (37°C and pH 7.4). The cuts were fitted with jointed thread clamps and put under 2 g of tension. At the same time, the aortic tissue in the Krebs solution was oxygenated by an oxygenating machine of 95% O_2_ and 5% CO_2_. Before recording the main contraction curve, the Krebs solution was replaced in the device three times every 5 min. After 15 min, the aortic contraction remained stable at the same 2‐g tensile strength. To measure the aorta contraction and relaxation, KCl (60 mM) solution was added to the Krebs solution after 15 min, and the aorta contraction was measured for 5 min (Qin et al., 2014).

### 2.7 Estimation of blood pressure parameters

A physiographic device was used to record the cardiac curve activity of rats. First, the rats were anesthetized using ketamine (0.013 mg/kg)/ xylazine (0.002 mg/kg) and put in the restrainer device. The cuff and sensor were then placed under the artery of rat tail bottom. The circulation of blood was blocked by the cuff. The blood reflowed and systolic and diastolic pressures were determined, while the rats were anesthetized. The heart activity chart of rats was drawn by a pen plotter in the direction of a crescent line on the physiographic paper (Kumar, Srivastava, Gupta, & Bajpai, 2017).

### 2.8 Statistical calculations

Statistical calculations were carried out by the ANOVA, and post hoc tests using SPSS 18 (*p* < .05). All values were reported as mean ± SEM (standard error of mean) (Al Batran et al., 2014). Data of negative and positive control group factors had no significant difference in the present study. Therefore, results of the negative control group were calculated as the control group of all analyses.

## 3 RESULTS

### 3.1 Rats' weights

Five‐month research on the complication of atherosclerosis: Changes in weight, serum lipids, and oxidative stress factors (MDA&ROS), histological aortic, contractile response of aorta (to 60 mM KCl), and changes in blood pressure parameters (systolic, diastolic, mean arterial, and pulse pressure) at male Wistar strain rats indicated that weights of experimental and sham groups, which received cholesterol 2% (through gavage) with a high‐fat diet (cholesterol 2%) for 40 days, did not increase significantly compared to control group at the end of a 40‐day period. After a 28‐day treatment, there were significant changes in weights of groups: Sham group, which daily received 1 mg/kg of saline water for 28 days, had the highest final weight compared to the control group (*p* < .05). The rats, which received atorvastatin and hydroalcoholic extract, had significantly lower final weights than the sham group (*p* < .05). The quercetin‐treated group had a significant decrease both in final weight and weight gain during the treatment compared to sham group (*p* < .01). Atorvastatin‐ and quercetin‐treated groups had total weight gain lower than the sham group (*p* < .05). There was no significant difference between experimental groups (except for the quercetin‐treated group) and control group. Final weight and weight gain during treatment had a significant decrease in the quercetin‐treated group compared to the control group (*p* < .05 and *p* < .01, respectively). The above two parameters in the quercetin‐treated group had a significant decrease compared to atorvastatin‐treated group (*p* < .05). Another weight parameter in quercetin‐treated group and all weight parameters in hydroalcoholic extract‐treated group had no significant difference with atorvastatin‐treated group (Table 1).

**Table 1:** Body weight comparison of rats. It indicates first weight, weight after 40 days, final weight, weight gain during treatment, and total weight gain means in five groups

Groups	Control	Sham	Atorvastatin (Treatment 1)	Quercetin (Treatment 2)	Hydroalcoholic extract (Treatment 3)
Parameters					
First weight (g)	179.6 ± 8.5	180.4 ± 10.1	180.9 ± 8.6	180.1 ± 9.8	179.4 ± 10.6
Weight after 40 days (g)	290.1 ± 8.1	303.9 ± 11.7	296.9 ± 11.8	300.1 ± 10.5	297.7 ± 14.3
Final weight (g)	352.6 ± 9.4	373.5 ± 12.7*	340.3 ± 14.3^#^	324.2 ± 11.8*^##a^	349.3 ± 11.1^#^
Weight gain during treatment (g)	62.5 ± 10.4	70.4 ± 15.0	43.4 ± 15.8	24.1 ± 3.7**^##a^	51.6 ± 17.6
Total Weight gain (g)	173 ± 13.8	193.1 ± 15.9	159.4 ± 12.9^#^	144.1 ± 16.9^#^	169.9 ± 15.6

Note

Data are expressed as means ± SEM. *p < .05 and **p < .01 compared to the control group. ^#^ *p* < .05 and ^##^ *p* < .01 compared to sham group. ^a^ *p* < .05 quercetin and hydroalcoholic extract‐treated groups compared to atorvastatin‐treatment group.

### 3.2 Serum lipids and blood oxidative stress factors

We measured serum lipids including the LDL, HDL, TC, TG, and blood oxidative stress factors including MDA and ROS (Table 2).

**Table 2:** Effects of atorvastatin, quercetin and hydroalcoholic extract on serum lipids and blood oxidative stress factors

Groups	Control	Sham	Atorvastatin (Treatment 1)	Quercetin (Treatment 2)	Hydroalcoholic extract (Treatment 3)
Parameters					
LDL (mg/dl)	74.52 ± 2.87	125.51 ± 12.49**	78.53 ± 2.05^##^	84.07 ± 3.84*^##a^	87.02 ± 6.14*^#a^
HDL (mg/dl)	67.35 ± 2.70	61.30 ± 2.53*	70.35 ± 7.54	81.10 ± 6.69*^#^	78.00 ± 5.22*^#^
TC (mg/dl)	85.43 ± 2.55	141.5 ± 13.10**	94.18 ± 3.90*^##^	89.64 ± 2.15^##^	101.5 ± 2.91*^#^
TG (mg/dl)	95.78 ± 5.8	130.96 ± 12.27*	108.00 ± 5.26*^#^	104.21 ± 6.09^#^	105.07 ± 7.32^#^
MDA (nmol/ml)	0.92 ± 0.01	1.81 ± 0.13**	1.15 ± 0.05*^##^	0.99 ± 0.05*^##a^	1.05 ± 0.04*^##a^
ROS (nmol/ml)	2.20 ± 0.30	4.60 ± 0.57**	3.46 ± 0.47*^#^	3.03 ± 0.32*^#^	3.10 ± 0.31*^#^

Note

Data are expressed as means ± SEM. * *p* < .05 and ** *p* < .01 compared to the control group. ^#^ *p* < .05 and ^##^ *p* < .01 compared to sham group. ^a^ *p* < .05 quercetin and hydroalcoholic extract‐treated groups compared to atorvastatin group.

Abbreviations: HDL, high‐density lipoprotein; LDL, low‐density lipoprotein; MDA, malondialdehyde; ROS, reactive oxygen species; TC, total cholesterol; TG, triglycerides.

Serum levels of LDL, TC, MDA, and ROS were significantly higher in the sham group than the control group (*p* < .01). Serum levels of TG also had a significant increase in this group compared to the control group (*p* < .05). Serum HDL level of sham group was significantly lower than the control group (*p* < .05). Serum HDL levels of quercetin and hydroalcoholic extract‐treated groups were significantly higher than sham and control groups (*p* < .05). This parameter was not significantly changed in atorvastatin‐treated group in comparison with the control and sham groups. Serum levels of LDL, TC, and MDA were significantly lower in atorvastatin and quercetin‐treated groups than the sham group (*p* < .01). Furthermore, serum levels of ROS and TG had a significant decrease in these two groups compared to the sham group (*p* < .05). Serum levels of TC, TG, MDA, and ROS in atorvastatin‐treated group and LDL, MDA, and ROS in quercetin‐treated group were significantly higher than the control group and serum levels of LDL, TC, MDA, and ROS in hydroalcoholic extract‐treated group (*p* < .05). Serum levels of LDL, TC, TG, and ROS were significantly lower in hydroalcoholic extract‐treated group than the sham group (*p* < .05). There was a significant decrease in serum MDA level of this group compared to the sham group (*p* < .01). Serum LDL level was significantly higher in quercetin and hydroalcoholic extract‐treated groups than atorvastatin‐treated group (*p* < .05). Moreover, there was a significant decrease in serum MDA levels of these two groups in comparison with the atorvastatin‐treated group (*p* < .05). Another parameter in quercetin and hydroalcoholic extract‐treated groups had no significant difference with atorvastatin‐treated groups (Table 2).

### 3.3 Histological studies

A transverse incision of the pectoral aorta as the involved major artery in atherosclerosis, shown with the colored pictures, was compared according to each of five groups (Figure 1). A marked increase in intima‐media thickness and fat accumulation was observed in sham group (B1 and B2 Textures, Figure 1). Large plaques were relatively created in the sham group (C2 Texture, Figure 1). The derived from the consumption of cholesterol 2% per 40 days with daily high‐fat food was mainly located at the intima layer of aorta and fully appeared in C2 texture photo. The treatment with atorvastatin, quercetin, and hydroalcoholic extract of *Otostegia persica* Boiss indicated that reduction of atorvastatin and quercetin increased intima‐media thickness and fat accumulation. They had better performance in cleansing plaque and atherosclerosis than the hydroalcoholic extract (D1, D2 and E1, E2, and F1, F2 Textures, Figure 1). These findings were obtained in rats after 28 days of treatment (Figure 1).

### 3.4 Tissue bath and physiography parameters

We measured tissue bath parameters (aorta contraction (g) and relaxation (%)) (Table 3) and physiography parameters including systolic pressure (mmHg), diastolic pressure (mmHg), mean arterial pressure (mmHg), and pulse pressure (mmHg) (Table 4).

**Table 3:** Comparison of aorta contraction (g) and relaxation (%) in five groups containing control, sham, atorvastatin, quercetin, and hydroalcoholic extract

Groups	Control	Sham	Atorvastatin (Treatment 1)	Quercetin (Treatment 2)	Hydroalcoholic extract (Treatment 3)
Parameters					
Aorta contraction (g)	0.34 ± 0.01	0.81 ± 0.12**	0.22 ± 0.02*^###^	0.26 ± 0.03*^##^	0.36 ± 0.02^##a^
Aorta relaxation (%)	21.6 ± 2.57	63.0 ± 4.27**	16.0 ± 2.71*^##^	17.62 ± 1.97*^##^	18.5 ± 2.25^##^

Note

Data are expressed as mean ± SEM. *p < .05 and **p < .01 compared to control group. ^#^ *p* < .05, ^##^ *p* < .01 and ^###^ *p* < .001 compared to sham group. ^a^ *p* < .05 quercetin and hydroalcoholic extract‐treated groups compared to atorvastatin group.

**Table 4:** Comparison of blood pressure parameters in five groups containing control, sham, atorvastatin, quercetin, and hydroalcoholic extract‐treated groups

Groups	Control	Sham	Atorvastatin (Treatment 1)	Quercetin (Treatment 2)	Hydroalcoholic extract (Treatment 3)
Parameters					
Systolic pressure (mmHg)	109.37 ± 4.17	177.50 ± 7.07**	119.37 ± 4.95*^##^	114.37 ± 4.17^##^	118.75 ± 7.9*^##^
Diastolic pressure (mmHg)	60.00 ± 3.77	81.87 ± 10.66*	73.12 ± 8.42*	63.75 ± 3.53^#^	65.62 ± 7.28^#^
Mean arterial pressure (mmHg)	76.62 ± 3.74	105.67 ± 15.3*	86.40 ± 2.59^#^	78.47 ± 2.58^#a^	83.10 ± 6.33^#^
Pulse pressure (mmHg)	50.00 ± 2.67	95.62 ± 11.78**	46.25 ± 5.17^##^	52.50 ± 5.97^##^	50.00 ± 6.54^##^

Note

Data are expressed as mean ± SEM. *p < .05 and **p < .01 compared to the control group. ^#^ *p* < .05 and ^##^ *p* < .05 compared to sham group. ^a^ *p* < .05 compared to atorvastatin group.

**Figure 2:**
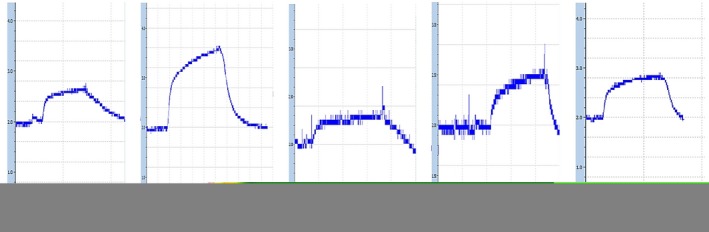
Presenting of the typical recording (tissue bath curves) of vasorelaxant effects of atorvastatin, quercetin and hydroalcoholic extract in three experimental group compared to control and sham groups. Vertical and horizontal bars indicate the force of aorta contraction (g) and the time (min) respectively

**Figure 3:**
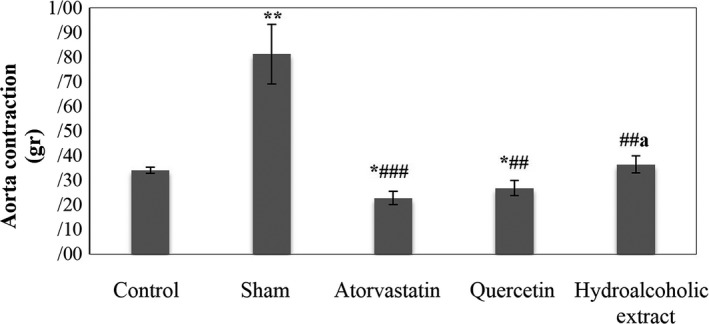
Aorta contraction. Data are expressed as means ± SEM. *p < .05 and **p < .01compared to control group. ^##^ *p* < .01 and ^###^ *p* < .001 compared to sham group. ^a^ *p* < .05 compared to atorvastatin‐treated group

**Figure 4:**
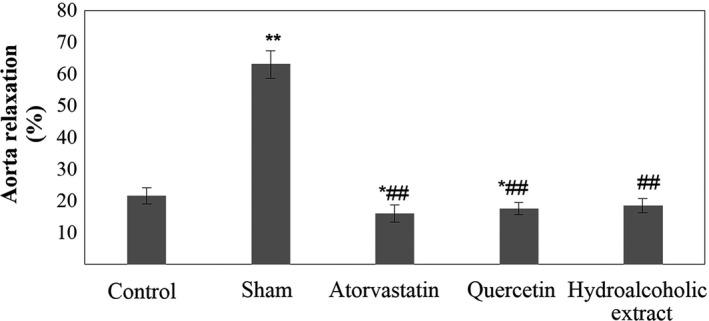
Aorta relaxation. Data are expressed as means ± SEM. *p < .05 and **p < .01 compared to control group. ^##^ *p* < .01 compared to sham group

Sham group had a significant increase in aorta contraction (contractile response of isolated aorta) and aorta relaxation compared to the control group (*p* < .01). Atorvastatin, quercetin, and hydroalcoholic extract‐treated groups had a significant decrease in aorta relaxation compared to the sham group (*p* < .01). This parameter and aorta contraction in atorvastatin and quercetin‐treated groups had a significant decrease in comparison with the control group (*p* < .05). There was a significant reduction in aorta contraction in the atorvastatin‐treated group compared to the sham group (*p* < .001). In quercetin and hydroalcoholic extract‐treated groups, aorta contraction was significantly lower than the sham group (*p* < .01). In addition, the quercetin‐treated group had no significant difference in aorta contraction and relaxation compared to the atorvastatin‐treated group. Hydroalcoholic extract‐treated group had no significant difference in aorta relaxation compared to the atorvastatin‐treated group. In this group, aorta contraction was significantly higher than atorvastatin‐treated group (*p* < .05) (Table 3) (Figures 2, 3, 4).

**Figure 5:**
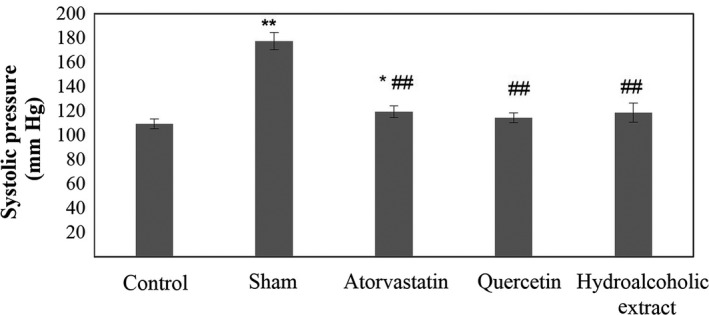
Systolic pressure in five groups. Data are expressed as means ± SEM. *p < .05 and **p < .01 compared to control group. ^##^ *p* < .01 compared to sham group

**Figure 6:**
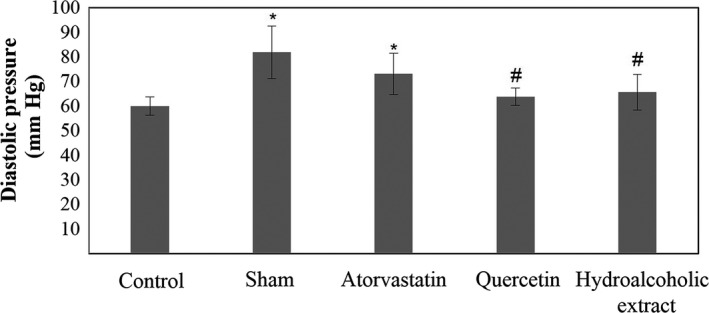
Diastolic pressure in five groups. Data are expressed as means ± SEM. *p < .05 compared to Control group. ^#^ *p* < .05 compared to sham group

**Figure 7:**
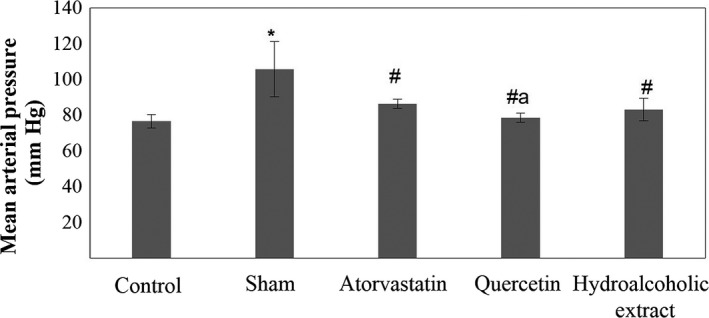
Mean arterial pressure in five groups. Data are expressed as means ± SEM. *p < .05 compared to control group. ^#^ *p* < .05 compared to sham group. ^a^ *p* < .05 compared to atorvastatin‐treated group

**Figure 8:**
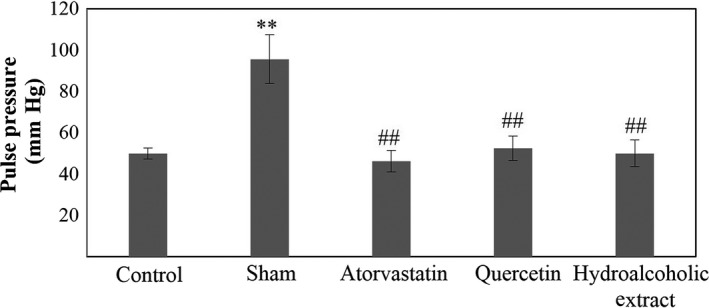
Pulse pressure in five. Data are expressed as means ± SEM. **p < .01 compared to control group. ^##^ *p* < .01 compared to sham group

Systolic and pulse pressure of sham group was significantly higher than the control group (*p* < .01). Diastolic and mean arterial pressure of this group had a significant increase in comparison with the control group (*p* < .05). Systolic and pulse pressure had a significant decrease in atorvastatin, quercetin, and hydroalcoholic extract‐treated groups (experimental groups) compared to the sham group (*p* < .01). Furthermore, mean arterial pressure of three groups had a significant decrease in comparison with the sham group (*p* < .05). There was no significant difference in diastolic pressure in the atorvastatin‐treated group compared to the sham group. This parameter was significantly lower in quercetin and hydroalcoholic extract‐treated groups than the sham group (*p* < .05). Systolic and diastolic pressure of atorvastatin‐treated group was significantly higher than the control group and also systolic pressure in hydroalcoholic extract‐treated group (*p* < .05). Another blood pressure parameter in three experimental groups had no significant difference with the control group. All blood pressure parameters of quercetin‐treated group (except for mean arterial pressure) and hydroalcoholic extract‐treated group had no significant difference with the atorvastatin‐treated group. There was a significant decrease in mean arterial pressure of quercetin‐treated group in comparison with the atorvastatin‐treated group (*p* < .05) (Table 4) (Figures 5, 6, 7, 8).

## 4 DISCUSSION

This study aimed to investigate comparative effects of quercetin and hydroalcoholic extract of otostegia persica Boiss with atorvastatin on atherosclerosis complication in male Wistar rats. Our data revealed that serum levels of LDL, TC, MDA, and ROS were significantly lower in experimental groups than sham group. In addition, there was a significant decrease in serum MDA levels of these two groups in comparison with the atorvastatin‐treated group. Blood pressure parameters were significantly decreased in treated rats with quercetin and hydroalcoholic extract in comparison with the sham group. These findings could present a beneficial treatment way for atherosclerosis disease. Atherosclerosis is characterized by increase in the thickness of intima‐media layer of artery, and it is the onset of cardiovascular diseases (Tatami et al., 2014). In the present study, a 40‐day gavage cholesterol 2% and high‐fat diet was used to cause atherosclerosis and create atheroma plaque in male Wistar strain rats. After 40 days, the plaque formation was clearly detected in intima layer of aorta regions near the heart. Moreover, we observed the destruction of endothelial layer along with an increase in artery wall thickness and accumulation of lipid layer in aorta of hypercholesterolemic rats. Earlier studies confirmed that high‐fat foods along with cholesterol at 6 to 12 weeks produced plaque and also increased body weights of rats (Panth et al., 2016). Results of the present study also confirmed the plaque formation after the consumption of cholesterol 2% per 40 days (through gavage) with high‐fat food daily intake. In addition, final weights of sham group rats significantly increased compared to the control group. Furthermore, serum levels of LDL, total cholesterol, and triglyceride, as well as elevated plasma serum levels of oxidant enzymes such as ROS and MDA significantly increased in the sham group in comparison with the control group.

Plaque formation in the intima layer is a complex process. Local hypercholesterolemia activates endothelial cells in arteries and retains lipoprotein, especially with LDL. This is the main factor in the process of formation of atheroma at the intima layer. The inhibition of fat accumulation, especially cholesterol within the cells and the connective tissue is the first step in improving or preventing atherosclerosis and the formation of plaque. The next step is to control the accumulation of smooth muscle cells along with variable amounts of inflammatory cells at the intima layer and lymphocytes in the vascular endothelium, oxidation of LDL through produced free radicals by macrophages, enzymatic changes in intima, and production of lipids, which are activated and effective in the formation of foam cells that produce cytokines (Sukhovershin, Furman, Tasciotti, & Trachtenberg, 2016).

In the present study, the above three experimental groups were separately treated with atorvstatin, quercetin, and hydroalcoholic extract of *Otostegia persica* Boiss in 28 days. In all three treatment groups, the thickness of vessel decreased, subendothelial fat layers reduced, and there was a lack of plaque formation, and internal lesions of the artery improved. This improvement was seen in a group of rats receiving quercetin and atorvastatin. We analyzed effects of these 3 products on several parameters including weight, serum lipids, and oxidative stress factors, aorta contraction, and blood pressure parameters. Histological studies were also conducted.

Results of the third group (compared to the sham group) indicated that the gavage of atorvastatin at 40 mg kg^‐1^ day^‐1^ improved the histological and biochemical parameters (serum lipids) and reduced levels of oxidative enzymes over 28 days. A significant decrease was observed in serum levels of TC, LDL, and TG, as well as ROS and MDA indicators. Moreover, the reduction in total weight gain was also significant and prevented the creation of atheromic plaque by decreasing levels of subendothelial fat and lipid oxidation. In Liyi Chi studies (2014), the use of atorvastatin in combination with berberine decreased TC, TG, and LDL in rats as well as the inflammation and oxidative stress. It also decreased endothelin‐1 levels (ET‐1).

The fourth group of rats received quercetin extract from the *Otostegia persica* Boiss plant. The main difference and advantage of quercetin‐receiving rats compared to atorvastatin‐treated group was a significant increase in serum HDL level and a significant decrease in serum MDA level compared to sham and control groups. This was associated with a significant decrease in LDL, TC, TG, and ROS levels compared to the sham group. This characteristic was apparent in final weight and weight gain during the treatment, so that they were significantly lower than the sham, control and atorvastatin‐treated groups.

Previous studies described that the obesity and hypercholesterolemia increased the production of free radicals and caused the oxidative stress. In fact, hypercholesterolemia is responsible for increasing the activity of oxidant enzymes and ultimately causes the cell damage. The activity of the enzyme in hypercholesterolemia increases ROS activity and leads to enhanced hydroxyl radicals. In addition, the increase of triglycerides causes an imbalance of oxidants and antioxidants, especially in obese rats (Azizova, Aseichev, Piryazev, Roitman, & Shcheglovitova, 2007). Due to the reduced levels of lipids and decreased lipid oxidation along with reduction in total weight gain, quercetin improved symptoms of histological atherosclerosis in aorta (isolated from male Wistar strain rats). The reduction of oxidative enzyme activity by quercetin was recorded during the Lucio G. Costa experiment (2016‐Hindawi Publishing Corporation) indicating a significant reduction in the ROS level compared to the inflammatory state (Costa, Garrick, Roque, & Pellacani, 2016). The findings of a research by Paulo Afonso Ribeiro Jorge et al. (2005‐Arquivos Brasileiros de Cardiologia journal) indicated a significant increase in LDL and cholesterol levels as well as plasma MDA and lipid oxidation and formation of atherosclerosis in the aorta (Jorge et al., 2005).

In the fifth group, the rats, which received the hydroalcoholic extract of the *O. persica* Boiss, had a significant increase in HDL levels (compared to the sham and control groups) as well as a significant decrease in LDL, TC, TG, MDA, and ROS levels compared to the sham group. Sedimentation and oxidation of cholesterol in arteries led to the vascular inflammation (Shrivastava, Singh, Raizada, & Singh, 2015). Cholesterol oxidation occurred mainly through a direct cholesterol interaction with ROS. High‐density lipoprotein ingredient played an important role in removing excess cholesterol from environmental tissues and transferring it to the liver for the elimination (Cruz, Mo, McConathy, Sabnis, & Lacko, 2013).

In this group, final weights of rats were significantly lower than the sham group, but had no significant difference with the control group. The results were consistent with previous findings. Findings of a research by Uchendu Ikenna Kingsley on "The effect of methanolic extract of Glycine max (soy bean) on high cholesterol diet‐fed albino rats" indicated a significant decrease in LDL, TC, TG and an increase in serum HDL levels (Kingsley et al., 2017). Furthermore, E.S. Omoregie found that methanolic extracts of certain Nigerian plants (plants included V. amygdalina) significantly reduced levels of ROS and MDA as an antioxidant (Omoregie & Osagie, 2012). This is likely the result of increased activity of ROS with higher level of MDA enzyme, stimulated inflammatory reactions, attracted blood monocytes, and mobilization and proliferation of macrophages derived from monocytes (Galkina & Ley, 2009). Cytokines along with macrophages, smooth muscle cells, and oxidized lipoproteins are effective in the creation of foam cells. Macrophages and smooth muscle cells devour oxidized lipoproteins and convert into foam cells that produce cytokines (Ramji & Davies, 2015). Activating the prevention of oxidative stress enzymes can also prevent atherosclerosis and ultimately prevent the formation of connective tissue by smooth muscle cells composing of collagen and elastic fibers and proteoglycans that are effective in the atherosclerosis (Badimon, Padro, & Vilahur, 2012).

In the present study, atorvastatin, quercetin and hydroalcoholic extract improved the atherosclerosis by reducing serum levels of LDL and cholesterol that was well documented in tissue samples (D1, D2 and E1, E2 and F1, F2 Textures respectively; Figure 1). Atorvastatin, quercetin and hydroalcoholic extract of *O. persica* Boiss significantly reduced levels of oxidative stress enzymes of ROS and MDA. We also observed reduced levels of oxidative enzymes in quercetin‐treated group along with an apparent increase in HDL levels, and a decrease in LDL serum levels.

With the accumulation of fat, the flexibility of artery was decreased and its interior space became narrow. The plaque was gradually formed in the inner wall of the artery and caused the tightness of blood vessel (Kallekar, Viswanath, & Anand, 2017). Hypercholesterolemia reduced the production and secretion of vasodilators deriving from endothelium and enhanced the generation of superoxide radicals in arteries. It also leads to atherosclerosis and results in increasing blood pressure by damaging endothelial cells of arteries. Atherosclerosis causes changes in the arterial wall including increase in their rigidity and contractility. It increases systolic, diastolic, mean arterial, and pulse pressure. By pressing arterial wall, high blood pressure leads to irreversible changes in the body's blood circulation system (Rafieian‐Kopaei et al., 2014).

Our findings indicated that the sham group had a significant increase in aorta contraction and systolic, diastolic, mean arterial, and pulse pressure compared to the control group. Results of contractile response of isolated aorta and blood pressure changes including systolic, diastolic, mean arterial, and pulse pressure in the experimental groups indicated that atorvastatin, quercetin and hydroalcoholic extract of *O.  persica* Boiss caused a significant decrease in aorta contraction. Quercetin and the hydroalcoholic extract led to a significant decrease in systolic, diastolic, mean arterial, and pulse pressure. Atorvastatin also led to a significant decrease in systolic and pulse pressure.

Ronaldo Altenburg Gismondi et al. found that anti‐hypertensive mechanisms of statins in reducing pulse rate resulting from lower arterial contraction due to the increased nitric oxide bioavailability increased endothelial‐dependent vasodilatation and decreased endothelin‐1 concentrations. In addition, they predicted that statins were RAAS (renin–angiotensin–aldosterone system) inhibitors that could block intracellular pathways associated with angiotensin II and could also reduce the expression of type 1 angiotensin receptors, and result in decreasing blood pressure (Gismondi et al., 2015).

High concentrations of extracellular KCl led to the depolarization of cell membrane, which increased the membrane permeability of Ca^2^⁺ in LVGC (L‐type voltage‐gated Ca^2^⁺ channels) opening, and then led to smooth muscle contraction of vessels. Flavonoids reduced blood pressure by decreasing the release of intracellular stored Ca^2^⁺ through voltage‐dependent Ca^2^⁺ channels (Qin et al., 2014).

In the present study, aorta relaxation time to reach the basal tone in sham group was longer than the control group and three experimental groups. Along with aorta contraction, the longer time was a major cause of pulse and high mean arterial pressure. In a research by Wang YK et al. (2009), the aorta relaxation was attributed to various reasons including decreased Ca^2^⁺ and increased K⁺ flux (Wang et al., 2009).

In a study by Susan WS et al. on high blood pressure in rats, the impact of components of hawthorn extract including flavonoids was reported as a regulating vascular tone. This effect was more significant in reducing diastolic pressure than systolic pressure (Susan, Wong, & Man, 2013). Their findings confirmed results of the present study on the vasoconstrictor tone and the flavonoid (quercetin and hydroalcoholic extract of *Otostegia persica* Boiss) effects on blood pressure parameters.

There are some limitations in the present study which should be mentioned. At first,....

## 5 CONCLUSION

Like atorvastatin, Quercetin and hydroalcoholic extracts of the *O.  persica* Boiss decreased serum lipids (except HDL), oxidative stress factors, and aorta contraction, weight gain, and blood pressure. They improved the vascular structure and prevented from the plaque formation by antioxidant activity. They were relatively more effective than atorvastatin in increasing serum HDL level and decreasing serum levels of MDA and could be considered as potential drugs in preventing atherosclerosis. These activities may be their relative advantages in comparison with atorvastatin in the treatment of atherosclerotic patients. Further research is necessary in this field. Limitations of this study are failure to measure the blood pressure parameters of the animal in consciousness, because for the systolic and diastolic pressure requires anesthetic and animal stimulation. Also, there were changes in aortic response to kcl over an hour from cutting to measurement.

## ETHICS STATEMENT

All experimental protocols, procedures and animals were reviewed and approved by Animal Care and Use Committee of the Faculty of Medicine and Health Sciences, Kashan University of Medical Sciences (IR.KAUMS.REC.1397.028).

## CONFLICT OF INTEREST

The authors of this work declare that they have no conflict of interest.

## References

[fsn31136-bib-0001] Agius, L. M. (2007). Complicated atheromatous plaque as integral atherogenesis. Journal of Clinical Pathology, 60(6), 589–592. 10.1136/jcp.2006.044107 17079352 PMC1955061

[fsn31136-bib-0002] Al Batran, R. , Al‐Bayaty, F. , Al‐Obaidi, M. M. J. , Hussain, S. F. , & Mulok, T. Z. (2014). Evaluation of the effect of andrographolide on atherosclerotic rabbits induced by Porphyromonas gingivalis. BioMed Research International, 2014, 1–11. 10.1155/2014/724718 PMC4151849 25215291

[fsn31136-bib-0003] Amna, B. , Uzma, S. , Umme, H. H. , Farha, A. , & Ambreen, M. U. (2016). Hyperlipidemia and hypertension; cardiovascular risk factors, various induction methods and their management by ethnomedicines. International Research Journal of Pharmacy, 7(11), 1–9. 10.7897/2230-8407.0711119

[fsn31136-bib-0004] Amom, Z. , Azman, K. F. , Ismail, N. A. , Shah, Z. M. , & Arshad, M. S. M. (2011). An aqueous extract of tinospora crispa possesses antioxidative properties and reduces atherosclerosis in hypercholesterolemic‐induced rabbits. Journal of Food Biochemistry, 35(4), 1083–1098. 10.1111/j.1745-4514.2010.00436.x

[fsn31136-bib-0005] Azizova, O. , Aseichev, A. , Piryazev, A. , Roitman, E. , & Shcheglovitova, O. (2007). Effects of oxidized fibrinogen on the functions of blood cells, blood clotting, and rheology. Bulletin of Experimental Biology and Medicine, 144(3), 397–407. 10.1007/s10517-007-0341-2 18457045

[fsn31136-bib-0006] Badimon, L. , Padro, T. , & Vilahur, G. (2012). Atherosclerosis, platelets and thrombosis in acute ischaemic heart disease. European Heart Journal: Acute Cardiovascular Care, 1(1), 60–74. 24062891 10.1177/2048872612441582 PMC3760546

[fsn31136-bib-0007] Costa, L. G. , Garrick, J. M. , Roque, P. J. , & Pellacani, C. . (2016). Mechanisms of neuroprotection by quercetin: Counteracting oxidative stress and more. Oxidative Medicine and Cellular Longevity, 2016, 1–10. 10.1155/2016/2986796. PMC4745323 26904161

[fsn31136-bib-0008] Cruz, P. M. , Mo, H. , McConathy, W. , Sabnis, N. A. , & Lacko, A. G. (2013). The role of cholesterol metabolism and cholesterol transport in carcinogenesis: A review of scientific findings, relevant to future cancer therapeutics. Frontiers in Pharmacology, 4, 119 10.3389/fphar.2013.00119 24093019 PMC3782849

[fsn31136-bib-0009] El‐Beltagi, H. S. , & Ahmed, M. M. (2016). Assessment the protective role of quercetin on acrylamide‐induced oxidative stress in rats. Journal of Food Biochemistry, 40(6), 715–723. 10.1111/jfbc.12262

[fsn31136-bib-0010] Fishbein, M. C. , & Fishbein, G. A. (2015). Arteriosclerosis: Facts and fancy. Cardiovascular Pathology, 24(6), 335–342. 10.1016/j.carpath.2015.07.007 26365806

[fsn31136-bib-0011] Galkina, E. , & Ley, K. (2009). Immune and inflammatory mechanisms of atherosclerosis. Annual Review of Immunology, 27, 165–197. 10.1146/annurev.immunol.021908.132620 PMC2734407 19302038

[fsn31136-bib-0012] Gismondi, R. A. , Bedirian, R. , Pozzobon, C. R. , Ladeira, M. C. , Oigman, W. , & Neves, M. F. (2015). Renin‐angiotensin system blockade associated with statin improves endothelial function in diabetics. Arquivos Brasileiros De Cardiologia, 105(6), 597–605. 10.5935/abc.20150123 26465872 PMC4693664

[fsn31136-bib-0013] Habauzit, V. , & Morand, C. (2012). Evidence for a protective effect of polyphenols‐containing foods on cardiovascular health: An update for clinicians. Therapeutic Advances in Chronic Disease, 3(2), 87–106. 10.1177/2040622311430006 23251771 PMC3513903

[fsn31136-bib-0014] Herrington, W. , Lacey, B. , Sherliker, P. , Armitage, J. , & Lewington, S. (2016). Epidemiology of atherosclerosis and the potential to reduce the global burden of atherothrombotic disease. Circulation Research, 118(4), 535–546. 10.1161/CIRCRESAHA.115.307611 26892956

[fsn31136-bib-0015] Insull, W. (2009). The pathology of atherosclerosis: Plaque development and plaque responses to medical treatment. The American Journal of Medicine, 122(1), 3–14. 10.1016/j.amjmed.2008.10.013 19110086

[fsn31136-bib-0016] Jones, P. H. , Davidson, M. H. , Stein, E. A. , Bays, H. E. , McKenney, J. M. , Miller, E. , … Blasetto, J. W. (2003). Comparison of the efficacy and safety of rosuvastatin versus atorvastatin, simvastatin, and pravastatin across doses. American Journal of Cardiology, 92(2), 152–160. 12860216 10.1016/s0002-9149(03)00530-7

[fsn31136-bib-0017] Jorge, P. A. R. , Almeida, E. A. D. , Ozaki, M. R. , Jorge, M. , & Carneiro, A. (2005). Effects of atorvastatin, fluvastatin, pravastatin, and simvastatin on endothelial function, lipid peroxidation, and aortic atherosclerosis in hypercholesterolemic rabbits. Arquivos Brasileiros De Cardiologia, 84(4), 314–319. 15880205 10.1590/s0066-782x2005000400008

[fsn31136-bib-0018] Kallekar, L. , Viswanath, C. , & Anand, M. (2017). Effect of wall flexibility on the deformation during flow in a stenosed coronary artery. Fluids, 2(2), 16 10.3390/fluids2020016

[fsn31136-bib-0019] Kim, M. H. , Lee, J. , Jung, S. , Kim, J. W. , Shin, J.‐H. , & Lee, H.‐J. (2017). The involvement of ginseng berry extract in blood flow via regulation of blood coagulation in rats fed a high‐fat diet. Journal of Ginseng Research, 41(2), 120–126. 10.1016/j.jgr.2016.01.004 28413315 PMC5386124

[fsn31136-bib-0020] Kingsley, U. I. , Steven, O. O. , Agu, C. E. , Orji, O. C. , Chekwube, B. E. , & Nwosu, T. F. (2017). Anti‐hyperlipidemic effect of crude methanolic extracts of Glycine max (soy bean) on high cholesterol diet‐fed albino rats. Journal of Medical & Allied Sciences, 7(1), 34–40. 10.5455/jmas.251532

[fsn31136-bib-0021] Kumar, P. , Srivastava, P. , Gupta, A. , & Bajpai, M. (2017). Noninvasive recording of electrocardiogram in conscious rat: A new device. Indian Journal of Pharmacology, 49(1), 116–118. 28458434 10.4103/0253-7613.201031 PMC5351223

[fsn31136-bib-0022] Kumar, S. , Kesari, K. K. , & Behari, J. (2010). Evaluation of genotoxic effects in male Wistar rats following microwave exposure. Indian Journal of Experimental Biology, 48(6), 586–592. 20882761

[fsn31136-bib-0023] Lorenz, M. W. , von Kegler, S. , Steinmetz, H. , Markus, H. S. , & Sitzer, M. (2006). Carotid intima‐media thickening indicates a higher vascular risk across a wide age range: Prospective data from the Carotid Atherosclerosis Progression Study (CAPS). Stroke, 37(1), 87–92. 10.1161/01.STR.0000196964.24024.ea 16339465

[fsn31136-bib-0024] Nadkarni, S. K. , Pierce, M. C. , Park, B. H. , de Boer, J. F. , Whittaker, P. , Bouma, B. E. , … Tearney, G. J. (2007). Measurement of collagen and smooth muscle cell content in atherosclerotic plaques using polarization‐sensitive optical coherence tomography. Journal of the American College of Cardiology, 49(13), 1474–1481. 10.1016/j.jacc.2006.11.040 17397678 PMC2785549

[fsn31136-bib-0025] Olia, J. B. H. , Ansari, M. H. K. , Yaghmaei, P. , Ayatollahi, H. , & Khalkhali, H. R. (2017). Evaluation of oxidative stress marker in patients with human papillomavirus infection. Annals of Tropical Medicine and Public Health, 10(6), 1518–1523. 10.4103/ATMPH.ATMPH_464_17

[fsn31136-bib-0026] Omoregie, E. , & Osagie, A. (2012). Antioxidant properties of methanolic extracts of some Nigerian plants on nutritionally‐stressed rats. Nigerian Journal of Basic and Applied Sciences, 20(1), 7–20.

[fsn31136-bib-0027] Panth, N. , Paudel, K. R. , & Parajuli, K. (2016). Reactive oxygen species: A key hallmark of cardiovascular disease. Advances in Medicine, 2016, 1–12. 10.1155/2016/9152732 PMC5059509 27774507

[fsn31136-bib-0028] Qin, X. , Hou, X. , Zhang, M. , Liang, T. , Zhi, J. , Han, L. , & Li, Q. (2014). Relaxation of rat aorta by farrerol correlates with potency to reduce intracellular calcium of VSMCs. International Journal of Molecular Sciences, 15(4), 6641–6656. 10.3390/ijms15046641 24747597 PMC4013652

[fsn31136-bib-0029] Rafieian‐Kopaei, M. , Setorki, M. , Doudi, M. , Baradaran, A. , & Nasri, H. (2014). Atherosclerosis: Process, indicators, risk factors and new hopes. International Journal of Preventive Medicine, 5(8), 927–946. 25489440 PMC4258672

[fsn31136-bib-0030] Ramji, D. P. , & Davies, T. S. (2015). Cytokines in atherosclerosis: Key players in all stages of disease and promising therapeutic targets. Cytokine & Growth Factor Reviews, 26(6), 673–685. 10.1016/j.cytogfr.2015.04.003 26005197 PMC4671520

[fsn31136-bib-0031] Rodriguez‐Saldana, J. , Rodriguez‐Flores, M. , Cantu‐Brito, C. , & Aguirre‐Garcia, J. (2014). A pathological study of the epidemiology of atherosclerosis in Mexico City. Cardiology Research and Practice, 2014, 1–8. 10.1155/2014/264205 PMC3955633 24719773

[fsn31136-bib-0032] Sadeghi, Z. , Akaberi, M. , & Valizadeh, J. (2014). *Otostegia persica* (Lamiaceae): A review on its ethnopharmacology, phytochemistry, and pharmacology. Avicenna Journal of Phytomedicine, 4(2), 79–88. 25050304 PMC4103708

[fsn31136-bib-0033] Safaeian, L. , Yaghoobi, S. , Javanmard, S. , & Ghasemi‐Dehkordi, N. (2017). The effect of hydroalcoholic extract of *Otostegia persica* (Burm.) Boiss. against H2O2‐induced oxidative stress in human endothelial cells. Research Journal of Pharmacognosy (RJP), 4(1), 51–58.

[fsn31136-bib-0034] Sanchis‐Gomar, F. , Perez‐Quilis, C. , Leischik, R. , & Lucia, A. (2016). Epidemiology of coronary heart disease and acute coronary syndrome. Annals of Translational Medicine, 4(13), 256–268. 10.21037/atm.2016.06.33 27500157 PMC4958723

[fsn31136-bib-0035] Sangkuhl, K. , Shuldiner, A. R. , Klein, T. E. , & Altman, R. B. (2011). Platelet aggregation pathway. Pharmacogenetics and Genomics, 21(8), 516–521. 10.1097/FPC.0b013e3283406323 20938371 PMC3134593

[fsn31136-bib-0036] Sharifi, N. , Mahernia, S. , & Amanlou, M. (2017). Comparison of different methods in quercetin extraction from leaves of *Raphanus sativus* L. Pharmaceutical Sciences, 23(1), 59–65. 10.15171/PS.2017.09

[fsn31136-bib-0037] Shrivastava, A. K. , Singh, H. V. , Raizada, A. , & Singh, S. K. (2015). C‐reactive protein, inflammation and coronary heart disease. The Egyptian Heart Journal, 67(2), 89–97. 10.1016/j.ehj.2014.11.005

[fsn31136-bib-0038] Sukhovershin, R. A. , Furman, N. E. T. , Tasciotti, E. , & Trachtenberg, B. H. (2016). Local inhibition of macrophage and smooth muscle cell proliferation to suppress plaque progression. Methodist DeBakey Cardiovascular Journal, 12(3), 141–145. 10.14797/mdcj-12-3-141 27826367 PMC5098570

[fsn31136-bib-0039] Susan, W. , Wong, M. , & Man, R. (2013). Effects of an extract of hawthorn on arterial blood pressure in anaesthetized rats. Cardiol Pharmacol, 2(1), 104–108.

[fsn31136-bib-0040] Tatami, Y. , Suzuki, S. , Ishii, H. , Shibata, Y. , Osugi, N. , Ota, T. , … Murohara, T. (2014). Impact of serum bilirubin levels on carotid atherosclerosis in patients with coronary artery disease. IJC Metabolic & Endocrine, 5, 24–27. 10.1016/j.ijcme.2014.08.006

[fsn31136-bib-0041] Tsoupras, A. , Lordan, R. , & Zabetakis, I. (2018). Inflammation, not cholesterol, is a cause of chronic disease. Nutrients, 10(5), 604 10.3390/nu10050604 PMC5986484 29757226

[fsn31136-bib-0042] Upadhyay, R. K. (2015). Emerging Risk Biomarkers in Cardiovascular Diseases and Disorders. Journal of Lipids, 2015, 1–50. 10.1155/2015/971453 PMC4407625 25949827

[fsn31136-bib-0043] Wang, Y.‐K. , Ren, A.‐J. , Yang, X.‐Q. , Wang, L.‐G. , Rong, W.‐F. , Tang, C.‐S. , … Lin, L. (2009). Sulfur dioxide relaxes rat aorta by endothelium‐dependent and‐independent mechanisms. Physiological Research, 58(4), 521–527. 18657003 10.33549/physiolres.931456

[fsn31136-bib-0044] Wang‐Michelitsch, J. , & Michelitsch, T. M. (2015). Misrepair mechanism in the development of atherosclerotic plaques. arXiv preprint arXiv:1505.01289.

[fsn31136-bib-0045] Yurdagul, A. , Finney, A. C. , Woolard, M. D. , & Orr, A. W. (2016). The arterial microenvironment: The where and why of atherosclerosis. Biochemical Journal, 473(10), 1281–1295. 10.1042/BJ20150844 27208212 PMC5410666

[fsn31136-bib-0046] Zarei, A. , Ashtiyani, S. C. , Taheri, S. , & Rasekh, F. (2014). Comparison between effects of different doses of Melissa officinalis and atorvastatin on the activity of liver enzymes in hypercholesterolemia rats. Avicenna Journal of Phytomedicine, 4(1), 15–23. 25050297 PMC4103723

